# Diagnostic Accuracy of Alvarado Versus Raja Isteri Pengiran Anak Saleha Appendicitis (RIPASA) Scores With Histopathology As Gold Standard for Acute Appendicitis: A Cross-Sectional Study

**DOI:** 10.7759/cureus.86151

**Published:** 2025-06-16

**Authors:** Raja Muhammad Mussab, Shehzadi Rimsha, Maria Kausar, Danish Aslam, Shehanshah Muhammed Arqam, Tehreem Ahsan, Hussain Ahmed Jhatial

**Affiliations:** 1 General Surgery, Dow University of Health Sciences, Civil Hospital Karachi, Karachi, PAK; 2 Orthopaedics and Trauma, Russells Hall Hospital, Dudley, GBR; 3 Trauma and Orthopaedics, Jinnah Postgraduate Medical Centre, Karachi, PAK; 4 General Surgery, Jinnah Postgraduate Medical Centre, Karachi, PAK

**Keywords:** acute appendicitis diagnosis, alvarado scoring system, diagnostic test accuracy, gold standard, histopathology (hp), ripasa score

## Abstract

Background: Diagnosing acute appendicitis is challenging in developing countries. The Alvarado and Raja Isteri Pengiran Anak Saleha Appendicitis (RIPASA) scoring systems have varying accuracies in diagnosing this condition. This study compares their diagnostic accuracy in our population.

Objective: To assess the diagnostic accuracy of Alvarado and RIPASA score for preoperatively diagnosing acute appendicitis, using histopathology as the gold standard.

Methods: A cross-sectional study was conducted from April 2021 to April 2022 at the Department of Surgery, Dr. Ruth K. M. Pfau Civil Hospital, Karachi. The estimated sample size was 187. The demographic and clinical characteristics of the patients were presented using frequency and percentage. Moreover, the chi-square test was applied to analyze the comparison between Alvarado and RIPASA scores for acute appendicitis. Sensitivity, specificity, and diagnostic accuracy were computed for both scoring systems. Histopathology reports served as the reference standard for diagnosis. The area under the Receiver Operating Curve (ROC) was determined for both Alvarado and RIPASA scores. A significance level of P value <0.05 was deemed statistically significant.

Results: Out of 187 participants, 82% (n=153) were diagnosed with appendicitis, comprising 125 male subjects and 62 female subjects. The mean age was recorded as 26.4 ± 8.5 years. The Alvarado score demonstrated sensitivity, specificity, and diagnostic accuracy of 93%, 31%, and 70%, respectively, while the RIPASA score showed sensitivity, specificity, and diagnostic accuracy of 83%, 36.3%, and 63%, respectively. The area under the ROC curve was 0.70 for the Alvarado score and 0.63 for the RIPASA scoring system.

Conclusion: In our study, the Alvarado score proved to be a more precise and sensitive diagnostic tool for identifying acute appendicitis in comparison to the RIPASA score. Due to its simplicity and reliable performance, the Alvarado score may be more practical to use where access to advanced diagnostic tools is restricted in resource-limited environments.

## Introduction

Acute appendicitis is one of the most common causes of acute abdominal pain, yet its diagnosis and management remain challenging [1]. Traditional symptoms and presentations account for only 60-70% of cases [2], leading to difficulties in timely diagnosis and treatment. Delays in management can result in complications such as perforation, especially [3]. Ahmed et al. reported an 11.6% prevalence of perforated appendicitis in tertiary care hospitals in Pakistan [4]. However, other studies reported varied rates of 14-16% with increased incidence in children and older populations [1]. To diagnose acute appendicitis effectively, a combination of clinical evaluation and laboratory investigations, including total leucocyte count, neutrophil count, and ultrasound, is essential [5]. The use of CT scans has improved diagnostic accuracy by up to 90%, with a reduction of negative appendectomies from 15-20% to 2-12% [3]. Despite these advancements, costly diagnostic facilities remain scarce in developing countries, making timely diagnosis challenging [6]. Access to advanced diagnostic tools is restricted in resource-limited settings; hence, the need for reliable and cost-effective diagnostic methods is critical. Scoring systems that integrate clinical history, physical examination, and laboratory results have been developed to facilitate the diagnosis of acute appendicitis [7]. The Alvarado scoring system, introduced in 1986, has been validated in various settings with sensitivity ranging from 53% to 88% [8,9]. Furthermore, the Raja Isteri Pengiran Anak Saleha Appendicitis (RIPASA) scoring system, incorporating additional parameters such as age, gender, and symptom duration [7,8], has demonstrated higher sensitivity (93.4%) and specificity (45.6%) [8]. Some studies favour the Alvarado system [6,10], while others find the RIPASA scoring system more reliable [7,9,11]. This study aims to assess the diagnostic accuracy of the Alvarado and RIPASA scores for the preoperative diagnosis of acute appendicitis at our hospital, using histopathology as the gold standard. The Alvarado score was developed based on Western populations, and studies have shown it may have lower sensitivity in South Asian patients, who often present later in the disease course and with atypical symptoms. In contrast, the RIPASA score was specifically designed for Asian populations and includes variables such as age, gender, and duration of symptoms, making it potentially more accurate in this setting.

The study seeks to identify which scoring system is more applicable to a developing country like Pakistan. Although our sample size and representation are limited to one hospital, the findings could offer valuable clinical implications and guide future research on scoring systems in Pakistan. This may help in potentially enhancing diagnostic precision and reducing negative appendectomies in resource-constrained settings.

## Materials and methods

This cross-sectional study was conducted from April 2021 to April 2022. Following approval from the Institutional Review Board, Sindh Government Hospital New Karachi (Approval No. SGHNK/1030), eligible patients who provided consent were enrolled at the Department of Surgery, Dr Ruth K. M. Pfau Civil Hospital, Karachi. A sample size of 187 cases, randomly selected, was calculated using the World Health Organization calculator [12] 95% confidence interval with an expected sensitivity of RIPASA score to be 96.7%, a specificity of 93.0% with a 6% margin of error with prevalence of disease as 58.05% [13].

The study included patients of both genders aged 13 and 50 years with clinical suspicion of acute appendicitis. Exclusion criteria comprised patients with appendicular lump or perforation, confirmed through clinical examination, pregnant women, recent abdominal trauma or previous abdominal surgery, pelvic inflammatory disease or history of urolithiasis, and non-consenting patients.

Upon admission, patients underwent a detailed history intake, including demographic information, followed by clinical examination to assess for signs of acute appendicitis. Complete blood count (CBC) and urine analysis were advised, and patients were assessed according to Alvarado and RIPASA scores [14]. Researchers completed the scoring charts at the time of admission. The Alvarado score contained a total of eight parameters, whereas the RIPASA score included 14 clinical parameters. In the Alvarado scoring system, a score of 7 or higher indicates a high probability of acute appendicitis, while for the RIPASA scoring system, a score of 7.5 or higher is considered indicative [15]. Patients having scores less than 7 for Alvarado and less than 7.5 for RIPASA were advised to have an ultrasound or CT scan. Ultrasound results were deemed positive if they revealed a blind-ending, non-compressible, non-peristaltic bowel loop arising from the cecum (appendix), the presence of an appendicolith, or a loculated para-caecal collection. Surgically excised specimens were collected using a non-probability consecutive sampling technique and were sent for histopathological examination after being fixed in 10% formalin. The specimens were processed at the Department of Pathology, Dow Medical College, Civil Hospital, Karachi. Histopathological findings of the operated cases were collected and compared with either score. A score ≥7 with a positive histopathology report was considered a true positive, while a negative histopathology report was a false positive. On the other hand, a score <7 with positive histopathology was considered to be a false negative, while negative histopathology was a true negative [13]. Histopathology was taken as the gold standard for diagnosis.

The data was collected and analysed using IBM SPSS Statistics for Windows, Version 23 (IBM Inc., Armonk, NY, USA). Quantitative variables were presented using mean and standard deviation, whereas qualitative variables used frequency and percentage. The chi-square test was applied for analysis of the comparison between the diagnostic accuracy of Alvarado and the RIPASA score for acute appendicitis. The sensitivity and specificity of Alvarado and RIPASA scores were also calculated. In addition, the area under the receiver operating curve (ROC) was calculated for the comparison of the diagnostic accuracy of Alvarado and RIPASA scores. P value < 0.05 was considered to be significant.

## Results

Out of 187 participants, (n=153) 82% were diagnosed to have appendicitis. In which, (n=125) 67% were males and (n=62) 33% were females. The mean age of patients was recorded as 26.4 ± 8.5 years. According to Table I, the RIPASA score, when applied to all the patients in the study, (n=176) 94% had a score ≥7.5, and only (n= 11) 5.9% had a score <7.5. Whereas more than half of the patients (n=100) had Alvarado Score ≥7, and (n=87) 46.5% had a score <7.

**Table 1:** : Distribution of patients in Alvarado and RIPASA scoring system RIPASA: Raja Isteri Pengiran Anak Saleha Appendicitis

	Alvarado		RIPASA	
Cut off	<7	≥7	<7.5	≥7.5
Frequency	87	100	11	176
Percentage	46.5	53.5	5.9	94.1

In accordance with Table 2, (n= 183) 98% had right Iliac fossa (RIF) pain, (n=159) 85% had anorexia, (n= 150) 80% had migratory RIF pain, (n=140) 75% had a duration of symptoms less than 48 hours, (n= 129) 69% had nausea and vomiting and only (n=34) 18% had a fever. Also, (n=173) 93% had RIF and rebound tenderness, and (n=172) 92.5% had guarding in RIF. Only (n=21) 11% of participants had Rovsing's sign present. (n=140) 75% had leucocytosis, (n=133) 71% had neutrophilia. Urine analysis came out to be negative for the majority of patients.

**Table 2:** Frequency distribution of clinical parameters RIF: right iliac fossa

Variable	Response	Frequency	Percentage
RIF pain	No	4	2
	Yes	183	98
Migratory RIF pain	No	37	19.8
	Yes	150	80.2
Anorexia	No	28	15
	Yes	159	85
Nausea and vomiting	No	58	31
	Yes	129	69
Duration of symptoms	<48 hours	140	74.9
	≥48 hours	47	25.1
Fever	No	153	81.8
	Yes	34	18.2
RIF tenderness	No	14	7.5
	Yes	173	92.5
Rebound tenderness	No	14	7.5
	Yes	173	92.5
Guarding in RIF	No	15	8
	Yes	172	92
Rovsing’s sign	No	166	88.8
	Yes	21	11.2
Neutrophilia	No	54	29
	Yes	133	71
Leukocytosis	No	47	25
	Yes	140	75
Urine analysis	Negative	161	86.1
	Positive	26	13.9

Chi-square test results in Table 3 show that (n=98) 98% of participants with an Alvarado score of ≥7 exhibited RIF tenderness (p=0.002). It implies a significant association between a higher Alvarado score and the presence of this clinical sign. Similarly, a significant association was noted between RIPASA score and rebound tenderness, where (n=170) 97% of participants with a RIPASA score of ≥7.5 showed rebound tenderness, while (n=8) 73% of participants with a score <7.5 did not (p=0.001). A further significant association was found between RIPASA score and guarding in RIF, with (n=170) 97% of those with a RIPASA score ≥7.5 exhibiting guarding and (n=9) 82% of participants with a score <7.5 showing no signs of guarding (p=0.0001).

**Table 3:** Comparison of Alvarado and RIPASA score with signs Comparison of Alvarado and RIPASA scores with clinical signs. Data are represented as n (%). Statistical significance was assessed using the chi-square test. A p-value < 0.05 was considered statistically significant. RIPASA: Raja Isteri Pengiran Anak Saleha Appendicitis; RIF: right Iliac fossa

Sign	Response	Alvarado <7 n (%)	Alvarado ≥7 n (%)	Alvarado χ²	Alvarado p-value	RIPASA <7.5 n (%)	RIPASA ≥7.5 n (%)	RIPASA χ²	RIPASA p-value
RIF tenderness	No	12 (14)	2 (2)	9.4	0.002	1 (9)	13 (7)	0.05	0.833
	Yes	75 (86)	98 (98)	9.4	0.002	10 (91)	163 (93)	0.05	0.833
Rebound tenderness	No	8 (9)	6 (6)	0.0	-	8 (73)	6 (3)	15.2	<0.0001
	Yes	79 (91)	96 (94)	0.0	-	3 (27)	170 (97)	15.2	<0.0001
Guarding in RIF	No	9 (10)	6 (6)	1.2	0.273	9 (82)	6 (3)	18.1	<0.0001
	Yes	78 (90)	94 (94)	1.2	0.273	2 (18)	170 (97)	18.1	<0.0001
Rovsing’s sign	No	79 (91)	87 (87)	0.7	0.40	11 (100)	155 (88)	1.5	0.223
	Yes	8 (9)	13 (13)	0.7	0.40	0 (0)	21 (12)	1.5	0.223

As per Table 4, when the chi-square test was applied, there was a correlation of Alvarado score with nausea and vomiting, in which (n=80) 80% of participants with Alvarado score ≥7 had nausea and vomiting (p= 0.0001).

**Table 4:** Comparison of Alvarado and RIPASA score with symptoms Comparison of Alvarado and RIPASA scores with symptoms. Data are represented as n (%). Statistical significance was evaluated using the chi-square test. A p-value < 0.05 was considered statistically significant. RIPASA: Raja Isteri Pengiran Anak Saleha Appendicitis; RIF: right Iliac fossa

Symptom	Response	Alvarado <7 n (%)	Alvarado ≥7 n (%)	Alvarado χ²	Alvarado p-value	RIPASA <7.5 n (%)	RIPASA ≥7.5 n (%)	RIPASA χ²	RIPASA p-value
RIF pain	No	1 (1)	3 (3)	0.77	0.382	0 (0)	4 (2)	0.26	0.611
	Yes	86 (99)	97 (97)	0.77	0.382	11 (100)	172 (98)	0.26	0.611
Migratory RIF pain	No	21 (24)	16 (16)	1.97	0.160	2 (18)	35 (20)	0.02	0.899
	Yes	66 (76)	84 (84)	1.97	0.160	9 (82)	141 (80)	0.02	0.899
Anorexia	No	17 (20)	11 (11)	2.42	0.120	3 (27)	25 (14)	1.40	0.237
	Yes	70 (80)	89 (89)	2.42	0.120	8 (73)	151 (86)	1.40	0.237
Nausea and Vomiting	No	38 (44)	20 (20)	15.6	<0.0001	6 (55)	52 (30)	3.0	0.081
	Yes	49 (56)	80 (80)	15.6	<0.0001	5 (45)	124 (70)	3.0	0.081
Duration of symptoms	<48 hours	64 (74)	76 (76)	0.11	0.744	7 (64)	133 (76)	0.79	0.374
	≥48 hours	23 (26)	24 (24)	0.11	0.744	4 (36)	43 (24)	0.79	0.374

The results of the chi-square test showed a correlation between histopathology and Alvarado score in which (n=93) 93% of participants with ≥7 Alvarado score were diagnosed with appendicitis and (n=60) 69% of participants with <7 Alvarado score did not have appendicitis (p= 0.0001) (Table 5).

**Table 5:** Comparison of Alvarado and RIPASA score with histopathology Comparison of histopathology results with Alvarado and RIPASA scores. Data are presented as n (%). Statistical significance was evaluated using the chi-square test. A p-value < 0.05 was considered statistically significant. RIPASA: Raja Isteri Pengiran Anak Saleha Appendicitis

Histopathology	Alvarado <7 n (%)	Alvarado ≥7 n (%)	Alvarado p-value	RIPASA <7.5 n (%)	RIPASA ≥7.5 n (%)	RIPASA p-value
Appendicitis	60 (69)	93 (93)	<0.0001	7 (64)	146 (83)	0.112
No appendicitis	27 (31)	7 (7)	-	4 (36)	30 (17)	-
Total	87	100	-	11	176	-

The chi-square test found a significant association between the Alvarado score and lab results (leucocytosis and neutrophilia). A total of (n=98) 98% of participants with an Alvarado score ≥7 had leucocytosis, while (n=45) 52% of those with a score <7 did not show leucocytosis (p=0.0001). Similarly, (n=81) 81% of participants with a score ≥7 had neutrophilia, whereas (n=35) 40% of those with a score <7 did not have neutrophilia (p=0.001) (Table 6).

**Table 6:** Comparison of Alvarado and RIPASA score with pathology Comparison of Alvarado and RIPASA scores with laboratory investigations. Data are presented as n (%). Statistical significance was determined using the chi-square test. A p-value < 0.05 was considered statistically significant. RIPASA: Raja Isteri Pengiran Anak Saleha Appendicitis

Investigation	Response	Alvarado <7 n (%)	Alvarado ≥7 n (%)	Alvarado χ²	Alvarado p-value	RIPASA <7.5 n (%)	RIPASA ≥7.5 n (%)	RIPASA χ²	RIPASA p-value
Leukocytosis	No	45 (52)	2 (2)	27.1	<0.0001	4 (36)	43 (24)	0.8	0.372
	Yes	42 (48)	98 (98)	27.1	<0.0001	7 (64)	133 (76)	0.8	0.372
Neutrophilia	No	35 (40)	19 (19)	10.5	0.001	6 (54)	48 (27)	3.1	0.051
	Yes	52 (60)	81 (81)	10.5	0.001	5 (46)	128 (73)	3.1	0.051
Urine analysis	Negative	76 (87)	85 (85)	0.22	0.641	7 (64)	154 (87)	5.4	0.020
	Positive	11 (13)	15 (15)	0.22	0.641	4 (36)	22 (13)	5.4	0.020

As per Table 7, the Alvarado score had a higher sensitivity at 93% but lower specificity at 31%. This means the Alvarado score has a greater ability to identify patients with acute appendicitis correctly but is less effective at ruling out those without the condition. In contrast, the RIPASA score had a slightly lower sensitivity of 83%, but its specificity was higher at 36.3%. This suggests that RIPASA may be more reliable in excluding non-appendicitis cases but with reduced sensitivity. The Alvarado score achieved a diagnostic accuracy of 70%, surpassing the 63% accuracy of the RIPASA score, making it the more accurate tool in this study. Both scoring systems have similar positive likelihood ratios (Alvarado 1.35, RIPASA 1.30), meaning that a positive test result moderately increases the likelihood of appendicitis. However, the Alvarado score has a lower negative likelihood ratio (0.226) compared to RIPASA (0.468), suggesting that a negative Alvarado score more effectively reduces the chance of appendicitis than a negative RIPASA score. In terms of predictive values, both scoring systems show comparable positive predictive values, which indicates that when the test is positive, there is a moderate probability that the patient has appendicitis. However, the Alvarado score has a higher negative predictive value (81.58%) compared to RIPASA (68.15%), suggesting that a negative Alvarado score is more reliable in ruling out the disease.

**Table 7:** Comparison between Alvarado and RIPASA scores RIPASA: Raja Isteri Pengiran Anak Saleha Appendicitis

Scoring system	Alvarado	RIPASA
Sensitivity (%)	93	83
Specificity (%)	31	36.3
Diagnostic accuracy (%)	70	63
Positive likelihood ratio	1.35	1.30
Negative likelihood ratio	0.226	0.468
Positive predictive value (%)	57.41	56.55
Negative predictive value (%)	81.58	68.15

**Figure 1:**
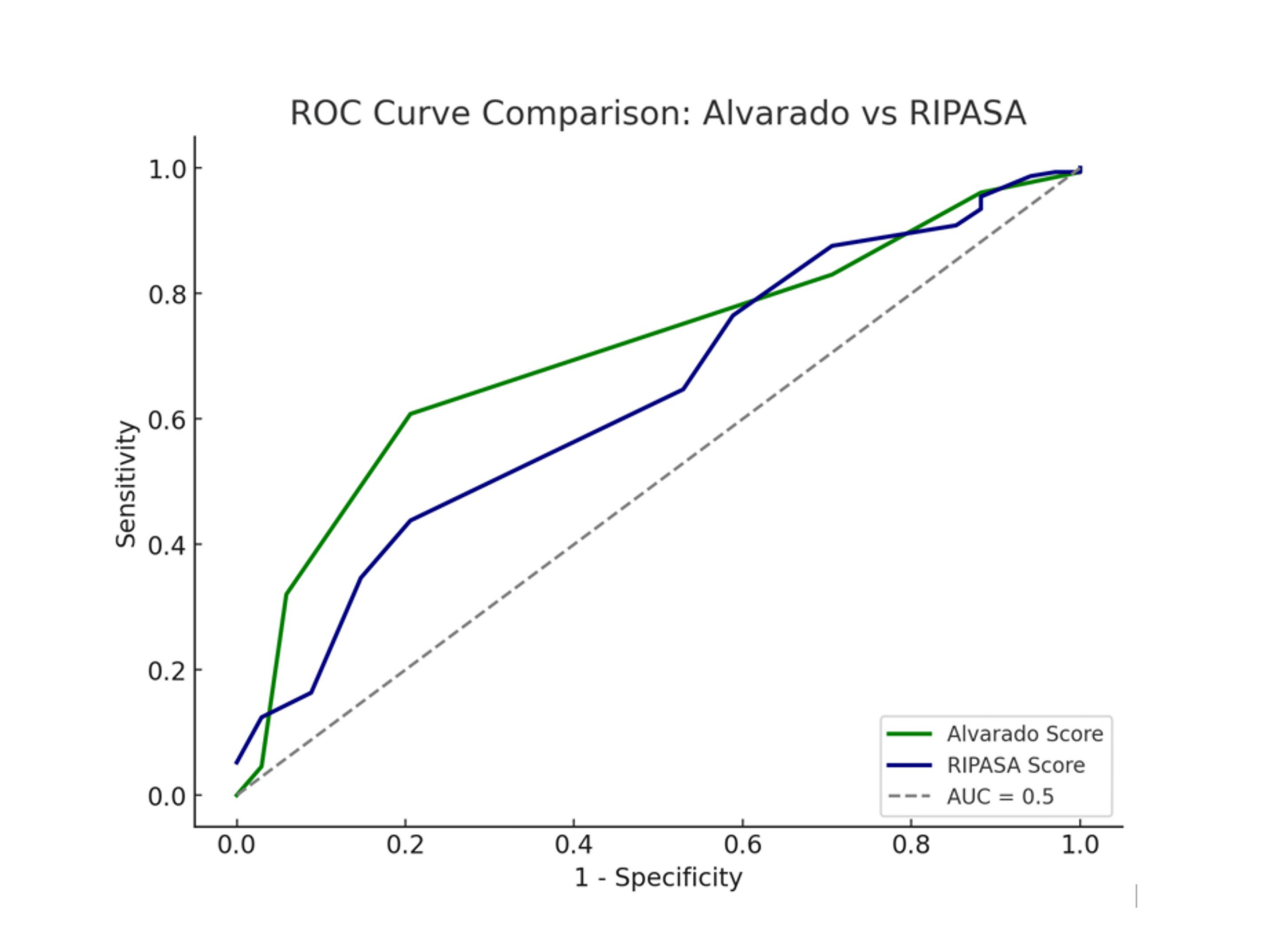
AUC for Alvarado versus RIPASA score RIPASA: Raja Isteri Pengiran Anak Saleha Appendicitis; ROC: receiver operating curve; AUC: area under the curve

Using ROC, the area under the curve (AUC) of RIPASA was 0.63 (Figure 1), and the Alvarado score was 0.70. Hence, a higher accuracy for the Alvarado score than the RIPASA score can be seen. The difference in AUCs was 7% (p-value: 0.001 vs. 0.0001).

## Discussion

As acute appendicitis manifests as a clinical constellation of symptoms which mimics many other abdominal conditions [7]. So clinical judgements are mostly supplemented by advanced radiological imaging like ultrasound with a sensitivity of 94.2% and specificity of 84.2% [10] and computed tomography scan with a much higher sensitivity and specificity of 97.6%, 83.3% and 96.6% diagnostic accuracy for acute appendicitis. On one hand, CT scans take the edge off the negative appendectomies [16], while on the other hand, it is one of the reasons for the delayed emergency appendectomies [8], which increases the risk of sepsis and perforation, which sequentially inflate the mortality and morbidity [7]. Moreover, the widespread utilization of CT scans may result in unnecessary appendectomies that could potentially be managed with antibiotics. Additionally, these investigations pose risks associated with radiation exposure, rely on operator proficiency, and necessitate radiologist interpretation, especially in cases with ambiguous findings [5]. Furthermore, in routine practices, these are not economic and serve to inflate the cost of healthcare, especially in countries with limited resources [11].

Thus, rapid and accurate diagnosis remains a difficult task in developing countries where clinical parameters remain the mainstay of diagnosis. Hence, a standard scoring system is needed to broaden the surgeon’s clinical assessment, the most important requisite to diagnose acute appendicitis quickly and affordably.

The results of our study concluded that the Alvarado scoring system is a relatively much better diagnostic scoring system for acute appendicitis than RIPASA. On the contrary, Chong et al. reported that the RIPASA score had better outcomes in Asian settings [9]. One possible explanation for the difference is the variation in patient demographics and healthcare settings. Our study focused on a population from Karachi, Pakistan, and Chong et al. had a sample from a hospital in Brunei Darussalam [9]. Moreover, our study is a prospective study, while Chong et al. is a retrospective study, which might account for the contrasting results [9].

Our study also revealed the excellent agreement between Alvarado and RIPASA score as 98% of participants were those who had ≥7.5 RIPASA score and ≥7 Alvarado score. Parallel to our findings, Regar et al. established a positive correlation between both scoring systems concerning acute appendicitis diagnosis [11]. Moreover, our study concluded the positive correlation of the Alvarado score with RIF tenderness and the RIPASA score with rebound tenderness and guarding in RIF. Similarly, Regar et al. and Flum et al. suggested that only RIF tenderness and rebound tenderness were found to be statistically significant [11,17].

According to our study, RIPASA was not significantly related to any symptom of acute appendicitis, whereas Alvarado score was correlated with nausea and vomiting. Our findings agreed with Korner et al., which also found nausea and vomiting statistically significant [18]. Conversely, the study by Regar et al. found anorexia out of all symptoms to be statistically significant [11].

In this study, we found a strong correlation between the diagnosis provided by the Alvarado scoring system and the histopathological acute appendicitis diagnosis. Similarly, Regar et al. reported both scoring systems are significantly associated with histopathological diagnosis [11].

Our study reported higher sensitivity (93%) but lower specificity (31%) of the Alvarado score compared to the RIPASA, with relatively higher specificity (36.3%) but lower sensitivity (83%). In resource-limited settings, the trade-off between high sensitivity and low specificity can significantly influence clinical decision-making. High sensitivity, like the 93% sensitivity of the Alvarado score observed in our study, reduces the likelihood of missed diagnoses, which is critical for conditions like acute appendicitis, where delayed treatment can lead to severe complications. However, the downside of this approach is its low specificity (31%), leading to a higher number of false positives. This may result in unnecessary surgeries or treatments, which can be especially problematic in settings where medical resources are scarce.

A study by Jang et al. revealed the lower sensitivity and specificity of the Alvarado score in diagnosing acute appendicitis when applied to the Asian population [19]. In addition, Nanjundaiah et al. reported higher sensitivity (96.2%) and specificity (90.5%) of the RIPASA score in the South Asian population, which is not consistent with our findings [7]. These differences may be linked to differences in study design, sample size, or the specific criteria used for each scoring system.

In accordance with the ROC of this study, the AUC for the Alvarado score (0.70) is relatively higher than the RIPASA score (0.63), representing the higher accuracy for the Alvarado scoring system. Whereas Nanjundaiah et al. revealed that the AUC of RIPASA was 0.982 and the Alvarado score was 0.849, which shows RIPASA had higher accuracy [7]. Likewise, Naz et al. suggested that the AUC of RIPASA was 0.962, and the Alvarado score was 0.938, which also shows the higher accuracy of the RIPASA score in the Asian population [20].

The study's strengths lie in its direct comparison of two widely used diagnostic scoring systems for acute appendicitis, particularly focusing on a South Asian population. By using histopathology as the gold standard, it provides a clear assessment of both the Alvarado and RIPASA score's accuracy. However, our study was limited to a single centre with a relatively small patient population to be considered as representative of the Pakistani population for choosing the Alvarado or RIPASA scoring system as a standard diagnostic tool for the conservative or surgical management of suspected cases of acute appendicitis. Therefore, further analysis from multicentre prospective studies is needed, particularly from developing countries of Asia, to reinforce the findings of our study.

In clinical practice, the findings suggest that the Alvarado scoring system may be more suitable for acute appendicitis diagnosis, particularly in resource-limited settings, where rapid and accurate decision-making is crucial. This has the potential to streamline patient management, optimize surgical interventions, and reduce the overall burden on healthcare resources in similar settings.

## Conclusions

In conclusion, our study found that the Alvarado scoring system is more effective than RIPASA in acute appendicitis diagnosis in a small sample in a Karachi, Pakistan setting. Given its simplicity and reliable performance, the Alvarado score may be more practical for use where access to advanced diagnostic tools is restricted in resource-limited environments. The Alvarado scoring system showed a significant correlation with histopathological acute appendicitis diagnosis.

The Alvarado scoring system’s parameters can easily be obtained through a comprehensive history, clinical examination, and two simple investigations, CBC and urine analysis. From an economic perspective, employing the Alvarado scoring system can reduce unnecessary inpatient admissions, costly radiological investigations, and unnecessary surgeries.
